# An Analysis of Clinical Outcomes of Exploratory Pediatric Metformin Ingestions Reported to the Texas Poison Center Network From 2011 to 2021

**DOI:** 10.1177/00185787241230628

**Published:** 2024-02-09

**Authors:** Shawn M. Varney, Sarah Watkins, Haylea Stuteville, Mark L. Winter, Han Tony Gao, Thomas G. Martin, Ryan P. Morrissey, Wayne R. Snodgrass, Brett A. Roth

**Affiliations:** 1 University of Texas Health San Antonio - San Antonio, TX, USA; 2 Texas Tech University Health Sciences Center El Paso, El Paso, TX, USA; 3 Texas Department of State Health Services, Austin, TX, USA; 4 University of Texas Medical Branch, Galveston, TX, USA; 5 Texas Tech University Health Sciences Center, Amarillo, TX, USA; 6 Baylor Scott & White Medical Center, Temple, TX, USA; 7 University of Texas Southwestern Dallas, Dallas, TX, USA

**Keywords:** drug information, medication safety, metabolic/endocrine, pediatrics, outcomes research, toxicology

## Abstract

**Background:** Poison centers develop triage threshold guidelines for pediatric metformin ingestions. Our network uses 1700 mg, or 85 mg/kg. **Objective:** To describe the dose, clinical course, and outcomes for inadvertent metformin ingestions in children 5 years old and younger reported to our statewide poison center network. **Methods:** We searched the poison center database 2011 to 2021 for metformin ingestions in patients 5 years and younger. Variables included age, sex, weight, dose, symptoms, outcome, and more. We used descriptive statistics with medians and interquartile ranges (IQR) for continuous variables. **Results:** Of 669 cases, exposures by age were 208 (31.1%) 1 to 2 years, and 275 (41.1%) 2 years. Weight was recorded in 342 (51.1%) (median 13.5 kg; IQR: 3.7 kg), and dose in 149 (22.3%) (median 500 mg; IQR: 500 mg). Milligram/kilogram values were available for 103 (15.4%) with median 42.4 mg/kg, IQR: 39 mg/kg. Most (647, 98.5%) exposures were unintentional. Most (445/669, 66.5%) were managed at a non-healthcare facility, while 204 (30.7%) were already at or referred to a healthcare facility. Of these 204 patients, 169 (82.8%) were evaluated and treated at the emergency department and discharged. Four (2%) were admitted to critical care, and 7 (3.4%) to the ward. Medical outcomes by effect were 5 (0.7%) minor, 2 (0.3%) moderate, 253 (37.8%) none, 292 (43.6%) not followed (minimal effects possible), and no major effects or deaths. Of 20 clinical occurrences reported, vomiting was most common (8, 1.2%). **Conclusion:** Despite little recorded dosage information, pediatric metformin ingestions under 85 mg/kg had predominantly uneventful medical outcomes.

## Introduction

Metformin (dimethylbiguanide) is an oral antihyperglycemic agent approved in 1996 for the management of type 2 diabetes mellitus. Its mechanism of action is to increase peripheral uptake and utilization of glucose and to decrease hepatic glucose production.^1,2^ Although hypoglycemia has been reported after a large intentional metformin overdose, metformin is not commonly expected to cause hypoglycemia in healthy patients.^3,4^ Its most serious adverse drug reaction is metformin-associated lactic acidosis (MALA), which may be life threatening, especially in elderly patients with chronic kidney disease.^5,6^

The Texas Poison Center Network is a group of 6 poison centers staffed by specialists in poison information (SPI) who follow approved guidelines to promote good medical outcomes. One purpose of poison center guidelines is to determine a safe threshold for keeping patients at home versus sending them to a healthcare facility for evaluation, potentially saving time, money, and resources. Safe triage thresholds are inherently difficult to determine due to the retrospective nature of human poisoning research. While previous studies have focused on adults suffering from MALA,^5,7,8^ children frequently ingest metformin, and caregivers may contact poison centers where SPIs provide recommendations based on experience and the limited amount of existing literature.^9,10^ Current triage thresholds for pediatric metformin ingestion are based on data from Spiller et al,^10^ who reported on metformin ingestion in 55 patients aged 15 months to 17 years, 44 of whom were 5 years old and younger. Based on these data, Spiller et al suggested that children who ingest less than 85 mg/kg, or 1700 mg (based on the 850 mg formulation available at that time), of metformin can be safely managed at home, while those who ingest more should be evaluated in a healthcare facility.

Our objective was to describe a larger cohort of patients 5 years old and younger with inadvertent, single-substance metformin ingestions and to identify the clinical course, outcomes, and milligram/kilogram (mg/kg) exposure reported to our statewide poison center network from 2011 to 2021. This information would provide a wider sense of the general safety of existing protocols using triage thresholds described by Spiller et al.^10^

## Methods

We searched the Texas Poison Center Network (TPCN) database (Toxicall^®^, v4.7.41, 1999-2013) for pediatric (5 years old and younger), single-substance metformin exposure cases for calendar years 2011 to 2021. Focus was placed on children 5 years old and younger because this age group is more likely to have inadvertent or exploratory ingestions compared to adolescents and older children, who may ingest metformin in large amounts to harm themselves. The product code, the most specific level of National Poison Data System (NPDS) substance coding hierarchy, identifies a specific substance, which often includes the brand name or formulation.^11^ The substance associated with the product code appears in the “substance description” field. We identified metformin exposures based on product codes. Data extracted included patient demographics (age, sex, and weight), relevant substance fields to determine amount ingested, clinical symptoms and signs, medical outcome, management site, and level of care at the healthcare facility using America’s Poison Centers definitions.^11^ Clinical effects that were related to the exposure or unknown if related were included as clinical findings.

We used the following available substance related fields in TPCN to calculate mg ingested: product code (specific for each metformin formulation/concentration), substance description, substance quantity (numerical quantity or amount for each substance) and quantity unit (see Appendix A), substance concentration and concentration unit (see Appendix A), substance dose and dose units (see Appendix A), and substance result (dose per weight based on calculation, ie, mg/kg).^11^ There were only 54 patients with a value for substance result; therefore, to maximize sample size for mg/kg analysis, the additional aforementioned substance fields were used to find all instances where amount ingested may have been captured to calculate mg. If the substance concentration, formulation, or dose unit was milligrams, then the numerical value in the corresponding field was used as “milligrams of substance.” The substance description field was searched for cases that did not explicitly capture milligrams ingested, and if milligram dose was included in the name, that was used as “milligrams of substance.” If the quantity units were “tabs/pills/capsules” or “each,” the “milligrams of substance” was multiplied by the substance quantity to get “substance dose (mg).” If the quantity units were “mg,” then the substance dose was the original “milligrams of substance.” In cases where both patient weight and substance dose were available, milligram/kilogram (mg/kg) was calculated, and the median dose and interquartile range were recorded. The Shapiro-Wilk test evaluated patient weight, substance dose, and mg/kg for normal distribution, and appropriate measures of central tendency were used to summarize the data. Cases that only had a “taste/lick/drop” of metformin were not included in the mg/kg calculations as the milligram ingested could not be determined for calculation. Cases with mg/kg were classified into the threshold categories—“safe” (less than or equal to 85 mg/kg) or “caution” (above 85 mg/kg)—to analyze current triage methods. Cases that only had a “taste/lick/drop” of metformin lacked information to determined milligram ingested but were included in the safe range, as it is reasonable to believe they would not have ingested enough to cause any effect.

Five cases with extended-release formulation metformin exposure were excluded from the dose calculation sub-analysis to minimize confounders as not enough data were available for comparison. Additionally, these 5 cases were not followed to clinical outcome, as they were expected to have only minimal clinical effects. We summarized the data with medians and standard deviations (SD) for continuous variables and percentages for dichotomous variables.

This study was approved by the University of Texas Health - San Antonio Institutional Review Board. The authors report no conflicts of interest.

## Results

**Figure 1.**
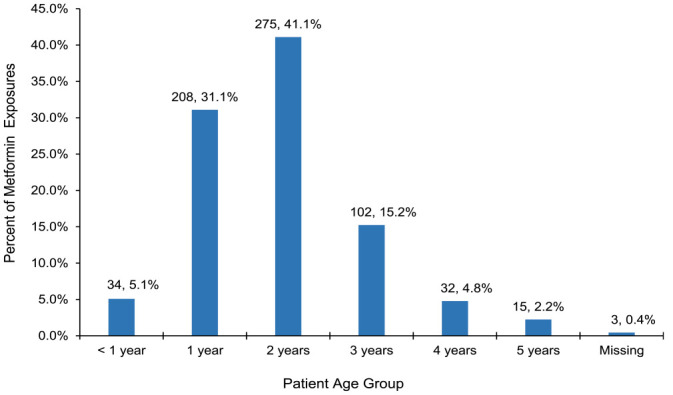
Number and percent of metformin exposure calls (N = 669) by patient age, 2011 to 2021.

There were 669 cases of single-substance metformin ingestion reported from 2011 to 2021, of which 391 (58.5%) were male, 271 (40.5%) female, and 7 (1%) unknown sex. Most (275, 41.1%) exposures occurred in toddlers aged 2 years old, followed by 208 (31.1%) patients 12 to 23 months of age (Figure 1).

All exposures were unintentional with an overwhelming majority (651, 99.1%) due to age-appropriate exploration. There was 1 case of unintentional misuse (0.1%) and 5 therapeutic errors (0.8%). Two-thirds (445, 66.5%) of cases were managed on site at a non-healthcare facility, while 175 (26.2%) were already in route to a hospital, 29 (4.3%) were referred to a hospital by the TPCN SPI, and 3% unknown location. (Table 1) Of the 204 cases already at, or referred to, a healthcare facility, 169 (82.8%) were evaluated and/or treated then released, and 11 (5.4%) were admitted (Table 2).

**Table 1.** Management Site of Calls to TPCN.

Management site (N = 681)	Number
Managed on site (non HCF)	445
Patient already in (enroute to) HCF when PCC called	175
Patient was referred by PCC to a HCF	29
Other	4
Unknown	28

*Note.* TPCN = Texas Poison Center Network; HCF = Healthcare Facility; PCC = Poison Control Center.

**Table 2.** Level of Care Received at a Healthcare Facility (HCF) for Calls That Were at, Enroute to, or Referred to a HCF.

Management at HCF (N = 204)	Number
Admitted to critical care unit	4
Admitted to noncritical care unit	7
Patient lost to follow-up/left AMA	21
Treated/evaluated and released	169
Referred to HCF: Patient refused referral/did not arrive at HCF	3

*Note.* AMA = against medical advice.

**Figure 2.**
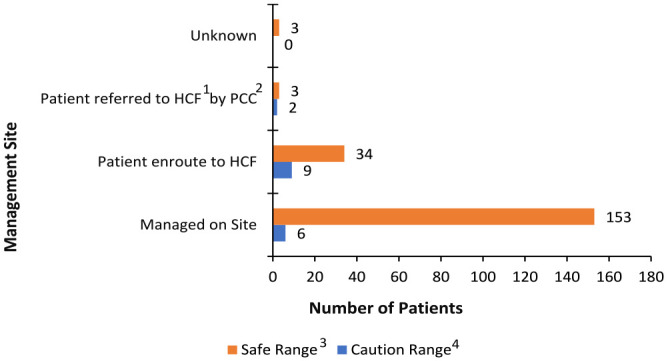
Management site of patients by milligram/kilogram threshold ranges, 2011 to 2021 (N = 210). ^1^HCF = Healthcare Facility. ^2^PCC = Poison Control Center. ^3^Safe range: less than or equal to 85 mg/kg metformin ingested. ^4^Caution range: greater than 85 mg/kg metformin ingested.

Patient weight, substance dose, and mg/kg were non-normally distributed (*P* < .0001). Half of cases (342, 51.1%) had a weight recorded (median 13.5 kg; IQR: 3.7 kg), while 149 (22.3%) had a metformin dose recorded (median 500 mg; IQR: 500 mg). Thus, mg/kg values were available for 103 (15.4%) cases (median 42.4 mg/kg; IQR 39 mg/kg). There were 109 patients who reported only a lick or taste of metformin. Of cases where mg/kg dosing was calculated, 193 (91.9%) were under the safe threshold (lick/taste included), and 17 (8.1%) in the caution range. Six (35.3%) cases in the caution range were managed on site instead of referred to a healthcare facility. Figure 2 shows the number of patients managed at different sites by threshold levels of caution and safety.

Twenty occurrences of clinical effects were reported in 16 patients among the 669 total: vomiting (n = 8), diarrhea (n = 1), hypoglycemia (n = 3), drowsiness/lethargy (n = 3), agitation/irritability (n = 1), seizure (n = 1), cough/choking (n = 1), diaphoresis (n = 1), and acidosis (n = 1). Most cases with recorded clinical effects in TPCN were missing data to calculate mg/kg, and were evaluated in, treated in, and released from an emergency department. The one patient with acidosis had a metformin exposure of 194.6 mg/kg. Activated charcoal was administered in 17 (8.3%) cases, 4 of whom were admitted, and the others were treated and released. Common treatments were to give food/water and to dilute/irrigate or wash the patient’s mouth.

Many exposures were judged by the poison specialists as inconsequential and expected to have no more than minimal clinical effects (612, 93.2%); therefore, they were not followed beyond the initial call. Further, of the 669 cases of ingestion, 7 (1.1%) resulted in a clinical finding that was minimally bothersome or just significant enough to require a simple intervention, 22 (3.3%) were lost to follow up and judged as potentially toxic, and 16 (2.4%) were recorded as non-exposure or unrelated effect. Twelve cases were missing and had no response recorded (Table 3).

**Table 3.** Medical Outcome of Pediatric Metformin Calls to TPCN.

Medical outcome (N = 669)	Number
Confirmed non-exposure	7
Minor effect	5
Moderate effect	2
No effect	253
Unable to follow, judged as a potentially toxic exposure	22
NF: minimal clinical effects possible (no more than minor effect possible)	292
NF: judged as nontoxic exposure (clinical effects not expected)	67
NF: Unrelated effect, the exposure was probably not responsible for the effect(s)	9
Missing	12

*Note.* TPCN = Texas Poison Center Network; NF = not followed.

## Discussion

This study represents the largest group of pediatric single-substance metformin ingestion cases recorded. Our study found minimal clinical effects overall, like previous results. In the study by Spiller et al., the median dose was 60 mg/kg, SD 40, like ours (median 42.4 mg/kg, SD 126.4). In their cohort, 67% were evaluated in a healthcare facility, and clinical effects were seen in 5 patients: nausea (n = 2) and dizziness (n = 1) manifested in 3 patients 6 years and older, whereas diarrhea (n = 2) occurred in 2 children 5 years and younger. In our study, in contrast to Spiller et al, two-thirds of patients were managed on site at a non-healthcare facility.

Among the 100 patients with documented mg/kg metformin dose, a dose of less than the 85 mg/kg threshold appeared to be safe for home monitoring of patients 5 years old and younger. Although all these patients were not followed to a known clinical outcome, there were no documented repeat calls by healthcare facilities or caregivers, and contact with them would exist over the decade the poison center was managing this common exposure. The lack of metformin adverse effects reported in MedWatch and in the America’s Poison Centers fatality reviews and the general medical literature also suggest that inadvertent metformin exposure in the pediatric population does not result in serious clinical effects. While the lack of evidence by itself does not support the contention of no/little risk, our study, in combination with the existing collective experience does. Until a change occurs in one of these reporting media, prospective studies seem unlikely since the adoption of the 85 mg/kg triage threshold by most US poison centers has already generally become a standard of care.

Although a more complete data set is desirable regarding the optimal safe mg/kg dose for metformin ingestion in children 5 years old and younger, additional studies would be difficult to perform given the standard that is currently practiced. Moreover, consumer safety reporting systems such as MedWatch have not identified metformin as a high-risk exposure in children. Furthermore, there are no cases of metformin-associated death in the fatality reviews for America’s Poison Centers for children with inadvertent metformin exposures, and review of published literature also shows no serious outcomes in our group of patients. The National Poison Data System data are less complete than our data but could potentially be included, although it would likely not yield additional information. Additionally, Poisindex^®^ states that ingestions of up to 1700 mg were well tolerated in healthy children.^12^ Finally, webPOISONCONTROL^®^ uses NPDS study data on 1300 children 5 years and younger with single-substance metformin ingestions with known mg/kg data. They found moderate outcomes in 87.5% with ingestions greater than or equal to 85 mg/kg, and only minor outcomes in 83.9% with ingestions less than 85 mg/kg. Clinical findings included hypoglycemia (24%), acidosis (22.6%), vomiting (13.3%), drowsiness (13.3%), coma (2.6%), seizures (1.3%), and renal failure (1.3%).^13^

Metformin is a remarkably safe medication with few adverse effects seen in this population of acute, unintentional ingestions. The anticipated serious outcome is MALA, defined by serum lactic acid >5 mmol/L and arterial pH < 7.5; however, MALA was not present in any of our study’s cases. Metformin associated lactic acidosis can occur at therapeutic doses in adults or in overdose, and it usually occurs in older patients with acute kidney injury or chronic kidney disease, with dehydration, and sepsis.^14,15^ It is rarely reported in children.

Of the 4 patients admitted to the pediatric intensive care unit, none had documented hypoglycemia, hyperlactatemia, or acidosis attributed to metformin. These findings are like Spiller et al^10^ and suggest an alternative cause. In our patients, only 1% of cases resulted in a medical outcome or clinical finding that was significant enough to require a simple intervention.^11^ The SPIs categorized most cases as inconsequential—either minimal clinical effects possible, or no effect. A significant limitation to our study is the lack of follow-up information for the 22 patients (3.3%) whose doses were considered potentially toxic but who were not able to be followed to outcome. Patients lost to follow up generally constituted this group.

Our study has several additional limitations. First, accurate determination of a toxic threshold for risk of serious clinical effects in children who inadvertently ingest metformin is difficult. Variables such as timing, pill counts, and actual versus perceived ingestion make data unreliable. Licks, sips, and tastes may be confused for actual ingestions, and tablets are often lost or missing for other reasons. Specialists in Poison Information must always consider the worst-case scenario; thus, what is recorded may be an overestimate. Our data also suggest a large percentage of patients presented to a healthcare facility without contacting the poison center first, so the mg/kg dose ingested was not available for triage purposes. In addition, we do not know the mg/kg dose in the 4.3% of patients whom the SPI referred to a healthcare facility. One would presume that the SPI who took the call and deemed the exposure “safe,” “not toxic,” or “minimal clinical effects possible,” would have obtained and recorded a patient weight and accurate dose information. Possibly the SPIs’ experience and lack of seeing adverse outcomes historically played a factor in the decision to keep the patient at home and/or to close the case. The National Poison Data System medical outcome field’s effect categories have objective parameters, but the decision is still subjective to the SPI and can be influenced by lack of obtaining pertinent information from the caller. Another limitation is the quality of documentation including the number of patients not followed to a known outcome, which can include NPDS categories like “not followed - no effects expected” and “not followed—minimal effects.” In cases of perceived nontoxic exposures, SPIs might select these categories based on their experience.

Although we do not have follow up for 60% of patients, a previously published report suggested that ingestions of 85 mg/kg would not result in toxicity in the pediatric population. Our poison center network’s 20 years of experience using this threshold value have shown that metformin ingestion at this dose is inconsequential. In addition, poison specialists routinely notify callers to call back with any problems or concerns. The lack of repeat calls from our patients and healthcare facilities supports the contention that they had an uneventful outcome. Additionally, close communication with surrounding hospitals exists and is a source of information for patients who were managed at home who presented to the hospital later. No such cases were identified in our database.

Texas Poison Center Network charts were often vague, missing information, or information was not captured in a consistent manner; for example, some only documented amounts as “1 tablet,” or “5 mL.” Cases that lacked explicit recorded dose estimated the dose in milligrams based on the name. Again, the SPIs’ experience with previous cases of little or no harm from ingestion of a single metformin tablet may have biased them not to record the details of the exposures. To overcome the challenge of collecting sufficiently detailed data through voluntary calls to the poison center, our statewide poison center network conducts monthly quality improvement assessments for various medications. We also select one focus area to measure and improve upon annually—for example, documenting the weight of children 12 years and younger, documenting specific dose and formulation of the medication ingested/inhaled/injected, documenting the correct units for drug concentrations, and following cases to conclusion of toxicity. These performance measures, along with continual education, emphasis, and reminders for our specialists in poison information, have improved documentation quality and compliance. As with most poison center data, it is likely that not all metformin exposures were reported to the poison centers since reporting is voluntary. Nevertheless, the absence of severe medical outcomes or clinical effects after metformin exposure in this study cohort over the 20 years we have used this threshold supports the continued practice of using 85 mg/kg as a triage threshold dose. The apparent rarity of significant toxicity in our population is somewhat reassuring. Cases with serious outcomes after inadvertent pediatric metformin should be reported.

## Conclusion

In this 10-year retrospective review of 669 children 5 years old and younger with unintentional single-substance metformin ingestion, we found very few adverse clinical effects. Although mg/kg data were limited in this cohort, a dose of up to 85 mg/kg appears to be a safe threshold for home monitoring.

## Appendix

**Appendix A.** Substance Quantity/Dose/Concentration Units.

mcg
mg
g
kg
ounces
lbs
mL
L
teaspoon
tablespoon
tabs/pills/capsules
taste/lick/drop
mouthful(s)
sip(s)
each (eg, bites/stings)
unknown
tab/tablet
ea/each
